# Evaluation of Constipation and Quality of Life Among Individuals Undergoing Hemodialysis: A Single-Center Cross-Sectional Study

**DOI:** 10.3390/healthcare13233095

**Published:** 2025-11-27

**Authors:** Asmaa Fatani, Reham W. Shafi, Hanadi Alhozali, Farouq Mohammad A. Alam, Abdulkader Monier Daghistani, Zulfitri Azuan Mat Daud, Bayan Tashkandi, Buthaina Aljehany

**Affiliations:** 1 Food and Nutrition Department, Faculty of Human Sciences and Design, King Abdulaziz University, Jeddah 22254, Saudi Arabia; btashkandi@kau.edu.sa (B.T.); baljehany@kau.edu.sa (B.A.); 2 Department of Clinical Nutrition, King Abdulaziz University Hospital, King Abdulaziz University, Jeddah 21589, Saudi Arabia; 3 Department of Nephrology, King Abdulaziz University Hospital, King Abdulaziz University, Jeddah 21589, Saudi Arabia; 4 Department of Statistics, Faculty of Science, King Abdulaziz University, Jeddah 21589, Saudi Arabia; fmalam@kau.edu.sa (F.M.A.A.); amadaghistani@kau.edu.sa (A.M.D.); 5 Department of Dietetics, Faculty of Medicine and Health Sciences, Universiti Putra Malaysia, Serdang 43400, Selangor, Malaysia

**Keywords:** hemodialysis, constipation, dietary fiber intake, quality of life, Saudi Arabia

## Abstract

**Aim:** To assess the prevalence of constipation, dietary fiber intake, and their association with QoL among HD patients. **Methods:** A cross-sectional study was conducted among 35 adults undergoing maintenance HD at King Abdulaziz University Hospital, Jeddah, Saudi Arabia. Constipation was assessed using the Rome IV criteria and Bristol Stool Form Scale (BSFS). QoL was evaluated using the Kidney Disease Quality of Life Short Form (KDQOL-36), and dietary fiber intake was estimated from three 24-h dietary recalls and a validated Dietary Fiber Intake Short Food Frequency Questionnaire (DFI-FFQ). Data were analyzed using Fisher’s exact test, the Mann–Whitney U test, and correlation coefficients (*p* < 0.05). **Results:** Constipation prevalence was 32%. Stool frequency was <3 per week in 14% of participants, 3–4 per week in 23%, and 7 per week in 63%. Mean fiber intake was 9.8 ± 4.8 g/day (24-h recall) and 8.6 ± 4.3 g/day (DFI-FFQ), with all participants classified as low fiber consumers. KDQOL-36 domain scores were highest for “effect of kidney disease” (80.4 ± 16.1) and lowest for “physical health” (40.4 ± 12.4). No significant associations were found between constipation, fiber intake, demographic or clinical variables, or any QoL domain (*p* > 0.05). **Conclusions:** Constipation is common among Saudi HD patients, yet not significantly linked to fiber intake or QoL in this small cohort. Larger multicenter studies are needed to identify determinants and guide evidence-based interventions.

## 1. Introduction

Constipation is a frequent gastrointestinal problem among patients with chronic kidney disease (CKD), particularly those undergoing maintenance hemodialysis (HD) [1]. Patients receiving HD represent a highly vulnerable clinical group, with markedly elevated morbidity and mortality risks. For example, the 1-year mortality rate after HD initiation reaches 15.4% in Japan—one of the countries with the lowest mortality rates globally—with infections and cardiovascular diseases being the leading causes of death [2]. Additionally, HD patients experience substantially lower QoL than the general population and kidney transplant recipients [3]. These vulnerabilities underscore the importance of managing modifiable symptoms—including constipation—that may further worsen clinical and psychosocial outcomes. Although it affects approximately 7.6% of the general population, studies indicate that constipation prevalence among HD patients can reach as high as 71.7% [4,5]. This high rate may be attributed to the use of constipation-inducing medications such as phosphate binders, fluid restriction, reduced physical activity, and dietary limitations [6]. Prolonged stool retention in the colon can increase stool firmness, cause abdominal discomfort, and lead to painful defecation, ultimately diminishing quality of life (QoL) [4,7]. Indeed, constipation is a significant factor known to adversely affect physical activity, comfort, mental well-being, and overall QoL scores [8,9]. Evidence shows that managing constipation can enhance the well-being and QoL of HD patients [10].

QoL is a multidimensional concept influenced by the physical, psychological, and social aspects of health, as well as subjective perceptions shaped by cultural contexts [11]. Assessing QoL among individuals with end-stage kidney disease is essential for evaluating the quality of care, guiding clinical decision-making, estimating community healthcare needs, and predicting patient prognosis [12,13]. Research has shown that HD patients generally have lower QoL scores than those undergoing peritoneal dialysis, especially in the physical and mental health domains [14]. This decline in QoL is linked to depression, recurrent infections, post-dialysis fatigue, and poor medication adherence [15]. Moreover, studies have demonstrated that interventions addressing constipation—such as elobixibat treatment and synbiotic supplementation—can significantly improve bowel symptoms and related QoL among HD patients [16,17].

Dietary fiber plays a central role in maintaining bowel regularity. However, HD patients often consume less fiber because high-fiber foods, such as fruits, vegetables, and legumes, are restricted to manage potassium and phosphorus levels [18]. Studies report that average fiber intake among HD patients typically ranges from 10 to 13 g/day, which is below recommended levels [19,20]. This insufficient intake may contribute to chronic constipation, yet few studies have investigated the link between fiber intake, constipation, and QoL in this group, with findings suggesting that higher fiber intake (especially from fruits) is associated with a lower risk of constipation [21]. Intervention trials using fiber supplements, such as polydextrose, have demonstrated improvements in bowel frequency and consistency in HD patients [22]. Hence, there is sparse research investigating how fiber intake may indirectly influence QoL in HD through alleviation of gastrointestinal symptoms, although in patients with chronic kidney disease more broadly (not restricted to HD), fiber supplementation has been linked to modest improvements in bowel function [23]. This gap in the literature highlights the need for further investigation into how dietary fiber intake interacts with constipation and QoL, specifically within HD populations.

Therefore, evaluating constipation, QoL, and fiber intake among HD patients is crucial to guide clinical practice, improve patient outcomes, and enhance overall well-being. Accordingly, this study aims to determine the prevalence and factors associated with constipation and QoL, with particular emphasis on the frequency of dietary fiber intake among individuals undergoing HD at King Abdulaziz University Hospital’s outpatient unit in Jeddah, Saudi Arabia. Based on the literature, we hypothesize that lower dietary fiber intake will be associated with a higher likelihood of constipation and that constipation will, in turn, be linked to lower QoL among HD patients.

## 2. Materials and Methods

### 2.1. Study Design and Participants

This cross-sectional observational study was conducted between December 2023 and January 2024 at a single HD facility in King Abdulaziz University Hospital, Jeddah, Saudi Arabia. Participants were eligible if they were aged 18 years or older, had been undergoing HD for at least three months, and were willing to participate. Patients who had started dialysis less than three months prior, were pregnant, or had recently undergone surgery were excluded. Eligible participants who agreed to take part signed an Informed Consent Form (ICF). During dialysis sessions, a registered dietitian explained the study objectives and the information to be collected. Participants were informed that participation was voluntary and that they had the right to withdraw at any time. The study protocol was approved by the Biomedical Ethics Research Committee of King Abdulaziz University (Reference No. 540-22).

**Figure 1:**
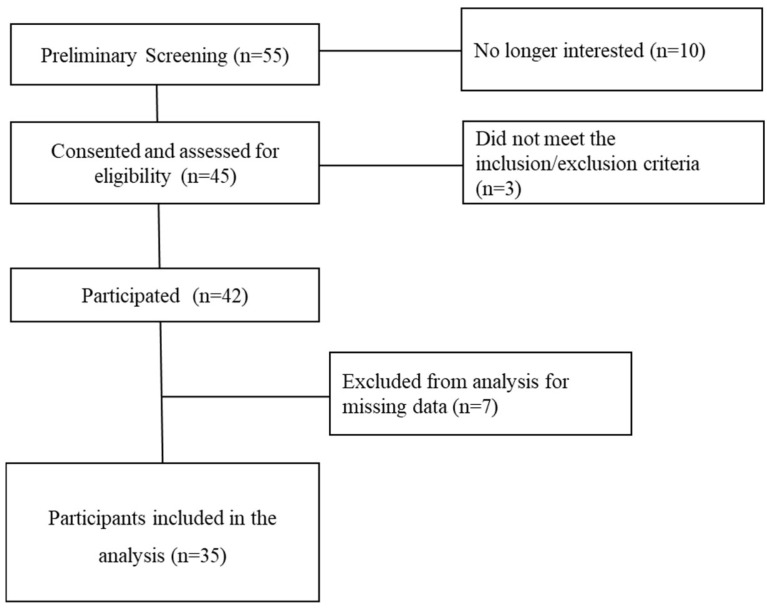
Participant recruitment and study completion.

A total of 55 individuals were initially screened. Of these, 10 lost interest, leaving 45 who consented and were assessed for eligibility. Among them, 3 did not meet the inclusion/exclusion criteria, resulting in 42 participants who took part in the study. During data processing, 7 participants were excluded due to missing data, leading to a final sample of 35 participants included in the analysis (Figure 1).

### 2.2. Data Collection

Data collection included obtaining sociodemographic and anthropometric information, as well as assessing constipation, quality of life (QoL), and dietary fiber intake. Sociodemographic data comprised age, gender, education level, marital status, and occupational status. Anthropometric measurements, including height and weight, were extracted from participants’ medical records, and body mass index (BMI) was calculated as weight in kilograms divided by height squared in meters (kg/m^2^).

Constipation was assessed using the Rome IV criteria and the Bristol Stool Form Scale (BSFS), both of which are standardized and validated tools widely used in clinical and research [24,25]. According to the Rome IV criteria, functional constipation is diagnosed when an individual presents with two or more of the following: straining during more than 25% of defecations, lumpy or hard stools (BSFS types 1 or 2) in more than 25% of defecations, sensation of incomplete evacuation in more than 25% of defecations, manual maneuvers to facilitate defecation in more than 25% of defecations, and fewer than three spontaneous bowel movements per week [26]. The BSFS is a descriptive, visual scale that classifies stool consistency into seven types ranging from hard or lumpy (Types 1–2) to loose or watery (Types 6–7) [27,28]. Participants were asked to indicate their most common stool type during the previous three months.

Quality of life was evaluated using the Kidney Disease Quality of Life Short Form (KDQOL-36), which is a reliable and validated tool for assessing the health-related QoL of dialysis patients. The Arabic validated version was used in this study [29]. This instrument covers five domains: symptom/problem list, effect of kidney disease, burden of kidney disease, physical health composite, and mental health composite. Scores for each domain range from 0 to 100, with higher scores indicating better QoL. Means and standard deviations were calculated for each domain and for the overall QoL score.

Dietary fiber intake was assessed using three 24-h dietary recalls and the Habitual Dietary Fiber Intake Short Food Frequency Questionnaire (DFI-FFQ). The three 24-h recalls were collected by the clinic dietitian, including one on a dialysis day, one on a non-dialysis day, and one on a weekend day. The Automated Self-Administered 24-Hour (ASA24^®^) tool was used to analyze dietary data. The DFI-FFQ, a validated instrument, was used to estimate the daily intake of dietary fiber (g/day) from five food groups: fruits, vegetables, bread and cereals, nuts and seeds, and legumes [30]. Participants reported their average consumption frequency using categories ranging from “never” to “six or more times per day.” Fiber content for each food group was calculated using the ASA24^®^ tool, and a scoring system was developed to classify participants as having low, moderate, or high dietary fiber intake. The classification cut-off points were as follows: for females, low: <18 g/day, moderate: 18–24.9 g/day, and high: ≥25 g/day; for males, low: <22 g/day, moderate: 22–29.9 g/day, and high: ≥30 g/day [31].

### 2.3. Statistical Analysis

Statistical analysis was performed using Excel and JASP 0.95.3 (a free and open-source statistical software for researchers). The analysis was bipartite. The first part of the analysis was descriptive; medians or means and standard deviations (SDs) were computed for numeric variables, while frequencies and percentages were calculated for categorical variables. The second part of the analysis was inferential, aiming to determine the association between demographic factors and dietary fiber intake with constipation prevalence and QoL. In addition, the association between constipation prevalence and QoL was assessed. All decisions were made based on a statistical significance threshold of *p* < 0.05.

Because the sample size was relatively small, some variables were adjusted, and explicit hypotheses were formulated to test associations. For instance, differences in dietary fiber intake between constipated and non-constipated participants (H_0_: no difference; H_1_: difference) were analyzed using the Mann–Whitney U test, as normality assumptions were not met. Similarly, the hypotheses were tested for BMI and age. To facilitate analysis, social status, occupational status, and education level were recoded as binary variables (married/not married, working/not working, university education/less than university education). Associations between constipation (binary outcome variable) and potential predictors were examined using binary logistic regression models. For each predictor variable, X, a separate model of the form

$$\log\left(\frac{P\left(Y=1\right)}{1-P\left(Y=1\right)}\right)=\beta_{0}+\beta_{1}X,$$

was fitted to estimate the odds ratio (OR) and its 95% confidence interval, where Y=1 indicates the presence of constipation. An OR greater than 1 indicates higher odds of constipation, while an OR less than 1 indicates lower odds.

For quality of life (QoL), associations with categorical variables were analyzed using the Mann–Whitney U test, while correlations with continuous variables (age, BMI, fiber intake) were determined using correlation coefficients. Given the small sample size, all analyses were considered exploratory, and no adjustments for multiple comparisons were applied.

Before discussing the statistical results, we computed Cronbach’s alpha to assess internal consistency according to item-level KDQOL-36 data. The estimated α = 0.587 (95% CI 0.293–0.882) indicated moderate internal consistency, which is reasonable given the small sample size (*n* = 35). Moreover, it is important to mention that this study included all eligible patients available during the data collection period, resulting in a total sample size of *n* = 35. Given the limited accessibility to the population and the exploratory nature of the research, a formal a priori power analysis was not conducted.

## 3. Results

### 3.1. Characteristics of the Study Participants

Thirty-five adults undergoing HD were recruited from the outpatient HD unit at King Abdulaziz University Hospital in Jeddah, SA. The characteristics of the study participants are presented in Table 1. The median age was 57 years, 63% were male, 40% had bachelor’s degrees, 57% were married, and 26% were employed. About half of the participants (48%) were either overweight or obese.

### 3.2. Fiber Intake

The fiber intake of study participants is presented in Table 2. The average of dietary fiber intake using 24-h dietary recalls was 9.8 ± 4.8 g/day; using the DFI-FFQ, the average of dietary fiber intake among study participants was 8.6 ± 4.3 g/day (Table 2). According to the cut-off points, all study participants were considered to have low dietary fiber intake.

### 3.3. Constipation

Table 3 indicates that most participants exhibited normal bowel function, with stool form and defecation frequency falling within healthy ranges. However, the presence of hard stools in 17% and low defecation frequency (<3 times per week) in 14% reveals the existence of constipation symptoms in these patients (Table 3).

### 3.4. Quality of Life

**Figure 2:**
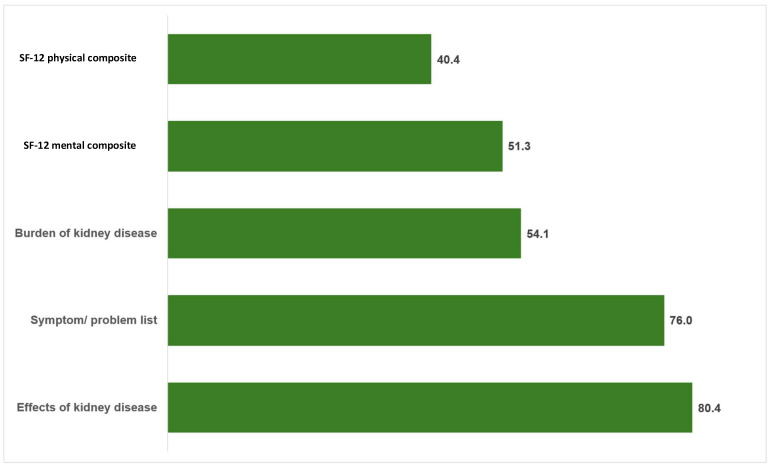
The mean score of KDQOL-36 domains among individuals undergoing hemodialysis (*n* = 35).

The mean scores of the KDQOL-36 domains—symptom/problem list, effect of kidney disease, burden of kidney disease, SF-12 physical health composite, and SF-12 mental health composite—were 76.0 ± 16.0, 80.4 ± 16.1, 54.1 ± 22.4, 40.4 ± 12.4, and 51.3 ± 10.7, respectively (Figure 2).

### 3.5. Association Between the Studied Parameters

**Figure 3:**
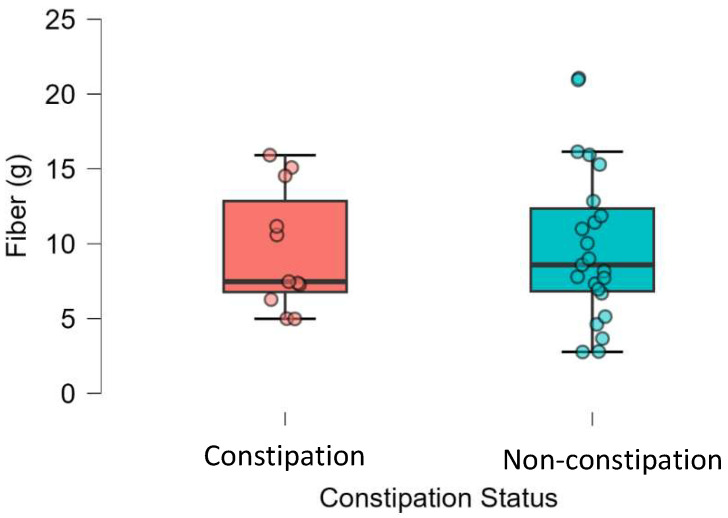
Dietary fiber intake among patients undergoing hemodialysis according to their constipation status (*n* = 35).

Figure 3 indicates that the non-constipation group has a wider range of fiber intake than the constipation group; thus, there was no significant difference between these two groups (*p* = 0.83). No significant associations were found between constipation and any of the examined variables. Specifically, logistic regression analysis indicated non-significant relationships with age (OR = 0.99, 95% CI 0.95–1.04, *p* = 0.73), gender (OR = 1.34, 95% CI 0.32–5.61, *p* = 0.69), educational level (OR = 2.75, 95% CI 0.59–12.85, *p* = 0.20), social status (OR = 0.64, 95% CI 0.16–2.63, *p* = 0.54), and BMI (OR = 1.00, 95% CI 0.86–1.16, *p* = 0.97). No significant associations were found between the total QoL score and participants’ age (*p* = 0.98), BMI (*p* = 0.98), gender (*p* = 0.78), educational level (*p* = 0.82), or social status (*p* = 0.40). There were no significant correlations between dietary fiber intake and any of the QoL domains, nor for the total score of QoL (*p* > 0.05) (Table 4). Likewise, no statistically significant association was observed between constipation and the overall QoL score (OR = 1.03, 95% CI 0.97–1.11, *p* = 0.29) (Table 5).

Moreover, no statistically significant associations were found between constipation and any of the KDQOL-36 domains. Logistic regression analyses indicated non-significant relationships with the symptom/problem list (OR = 1.04, 95% CI 0.99–1.09, *p* = 0.13), effect of kidney disease (OR = 1.01, 95% CI 0.96–1.05, *p* = 0.80), burden of kidney disease (OR = 1.01, 95% CI 0.98–1.04, *p* = 0.62), SF-12 physical health composite (OR = 1.04, 95% CI 0.98–1.10, *p* = 0.24), or SF-12 mental health composite (OR = 1.01, 95% CI 0.95–1.08, *p* = 0.79) (Table 6).

## 4. Discussion

This study examined the prevalence of constipation, dietary fiber intake, and their association with quality of life (QoL) among individuals undergoing maintenance hemodialysis (HD) at a tertiary hospital in Jeddah, Saudi Arabia. Constipation was prevalent in 32% of participants, and all individuals had low dietary fiber intake. However, no statistically significant associations were observed between constipation, sociodemographic characteristics, fiber intake, or QoL domains.

The 32% prevalence of constipation aligns with previous investigations reporting rates between 13.5% and 71.7% among HD populations [6,7,32,33]. The variation across studies likely reflects differences in diagnostic criteria, assessment tools, and population characteristics. In Saudi Arabia, [1] reported a higher constipation prevalence of 53% using the Rome IV criteria, possibly due to older age or longer HD duration among participants. Common contributors to constipation in HD patients include fluid restrictions, low physical activity, phosphate binder use, and dietary limitations [14,34].

In the current study, the average fiber intake was 9.8 g/day based on 24-h recalls and 8.6 g/day according to the DFI-FFQ, consistent with findings from other HD populations reporting intakes below the recommended 25–30 g/day [19,20]. Such low intake is expected, given dietary restrictions on potassium- and phosphorus-rich foods like fruits, vegetables, and legumes [35]. Despite low fiber intake being a recognized risk factor for constipation in the general population [18], no significant association was found in this study, possibly due to the uniformly low fiber consumption and small sample size.

The QoL findings indicated moderate-to-high scores across most KDQOL-36 domains, with the “effect of kidney disease” domain scoring the highest (80.4 ± 16.1) and “physical health composite” the lowest (40.4 ± 12.4). This pattern is consistent with previous research, where physical health was the most impaired domain among HD patients [15,36]. Reduced physical QoL among HD patients may result from treatment-related fatigue, comorbidities, and the chronic burden of therapy [14,37]. Conversely, higher scores in kidney-disease-specific domains may reflect adaptation and psychosocial support within the dialysis environment [12].

No significant association was found between constipation and any QoL domain in this study. While previous research has shown that constipation negatively affects QoL and psychological well-being in CKD populations [4,8], the absence of a relationship here could be due to the small sample size and possible adaptation among long-term HD patients. Furthermore, unmeasured factors such as depression, social support, and healthcare access might have influenced QoL more than constipation itself [38].

The present study has several limitations that should be discussed. First, the small sample size may impact the significance of associations and generalizability of the results. Furthermore, confounding variables need to be addressed to provide a more comprehensive understanding of the factors influencing QoL among HD patients. Recruiting participants undergoing HD was challenging, as many tend to sleep during dialysis sessions or lack the energy to engage in conversations. Furthermore, the questionnaires on constipation and QoL were self-reported, which may introduce bias due to inaccuracies or subjectivity in reporting. Moreover, the questionnaire used in this study to evaluate dietary fiber intake, the FFQ, was qualitative. Therefore, it could not quantify the accurate amount consumed in portions or grams per day. Addressing these limitations and including a larger, more diverse sample in future research would provide a more comprehensive understanding of the factors influencing QoL and constipation among HD patients.

## 5. Conclusions

Constipation was prevalent among participants undergoing HD, yet no significant association was observed with the quality of life in this population, likely reflecting the limited sample size. Overall, the results highlight the need for larger multicenter studies to clarify the determinants of constipation and QoL among HD patients in Saudi Arabia. Interventions promoting safe dietary fiber sources and physical activity could help mitigate constipation and enhance overall well-being in this population.

## Figures and Tables

**Table 1:** Characteristics of study participants (*n* = 35).

Characteristic	*n* (%)
**Sex**	
**Male**	22 (63)
**Female**	13 (37)
**Age (Years)**	Median (range) 57 (19–84)
**Education Level**	
**(Less than University Education)**	**16 (45.71)**
**Uneducated**	3 (8.57)
**Elementary School**	3 (8.57)
**Middle School**	3 (8.57)
**High School**	7 (20.00)
**(University Education)**	**14 (40.00)**
**Bachelor**	14 (40.00)
**(Higher Education)**	**5 (14.29)**
**Social Status**	
**(Married)**	**20 (57)**
**(Not Married)**	**15 (43)**
**Single**	9 (26)
**Divorced**	1 (3)
**Widowed**	5 (14)
**Occupation Status**	
**(Not Working)**	**26 (74)**
**Unemployed**	13 (37)
**Retired**	13 (37)
**(Working)**	**9 (26)**
**Employed**	7 (20)
**Free Business**	2 (6)
**BMI**	
**Underweight**	3 (9)
**Normal**	15 (43)
**Overweight**	11 (31)
**Obese**	6 (17)

**Table 2:** Fiber intake of participants undergoing hemodialysis (*n* = 35).

	Dietary Intake (Mean ± SD)
**Fiber—24-h dietary recalls (g)**	9.8 ± 4.8
**Fiber—DFI-FFQ (g)**	8.6 ± 4.3

**Table 3:** Type and frequency of stool among individuals undergoing hemodialysis (*n* = 35).

Type and Frequency of Stools		(%)
**Type of Stool**	Hard stool	17
	Normal stool	80
	Soft stool	3
**Frequency of Defecation**	<3 stools/week	14
	3–4 stools/week	23
	7 stools/week	63

**Table 4:** Correlation and association analyses between QoL and selected demographic/clinical characteristics.

Variable	*p*-Value
Age	0.98
BMI	0.98
Gender	0.78
Educational level	0.82
Social status	0.40

**Table 5:** Logistic regression analysis showing associations between constipation and selected demographic and clinical variables (*n* = 35).

Variable	OR (95% CI)	*p*-Value
Age (years)	0.99 (0.95–1.04)	0.73
Gender (male: X = 1)	1.34 (0.32–5.61)	0.69
Edu. level (uni. education: X = 1)	2.75 (0.59–12.85)	0.20
Social status (not married: X = 1)	0.64 (0.16–2.63)	0.54
BMI	1.00 (0.86–1.16)	0.97
Overall QoL score	1.03 (0.97–1.11)	0.29

**Table 6:** Association between constipation and KDQOL-36 domain scores (*n* = 35).

Variable	OR (95% CI)	*p*-Value
Symptom/problem list	1.04 (0.99–1.09)	0.13
Effect of kidney disease	1.01 (0.96–1.05)	0.80
Burden of kidney disease	1.01 (0.98–1.04)	0.62
SF-12 physical composite	1.04 (0.98–1.10)	0.24
SF-12 mental composite	1.01 (0.95–1.08)	0.79

## Data Availability

The datasets generated and/or analyzed during the current study are not publicly available as they contain sensitive personal information, but they are available from the corresponding author upon reasonable request.
